# Arachnoid diverticulum diagnosis following treatment of cryptococcal meningitis in a dog

**DOI:** 10.1002/ccr3.1325

**Published:** 2018-02-07

**Authors:** Evelyn Galban, Jennifer Perkins

**Affiliations:** ^1^ Department of Clinical Sciences and Advanced Medicine School of Veterinary Medicine University of Pennsylvania Philadelphia Pennsylvania; ^2^ Department of Neurology and Neurosurgery Pieper Memorial Veterinary Center Middletown Connecticut

**Keywords:** Amphotericin, arachnoid diverticulum, Cryptococcosis, *Cryptococcus* latex agglutination titer, fluconazole

## Abstract

Successful long‐term treatment of cryptococcal meningitis in dogs is clinically challenging. In humans, there are only rare case reports of complications post‐treatment including arachnoid diverticula. Combination antifungal therapy is standard of practice in human medicine and should be considered in veterinary patients.

## Case Presentation

An 11‐month‐old intact male Irish Setter (29 kg [63.8 lb]) presented to the Penn Vet Ryan Hospital at the University of Pennsylvania for acute onset of vestibular signs. The primary veterinarian noted a vestibular ataxia and nystagmus (no further characterization). The patient was administered cephalexin, carprofen, and meclizine and was transferred to the emergency service at Penn Vet.

On presentation, the patient was quiet but alert and responsive with a vestibular ataxia and right head tilt. A changing nystagmus (resting rotary fast phase left to vertical in dorsal recumbency) was noted. Proprioception was normal in all limbs with increased segmental reflexes in the pelvic limbs. A fundic examination was normal. Central vestibular signs were localized to the caudal fossa.

Hematologic and biochemistry panels were normal. Serology for *Rickettsia rickettsii*,* Neospora caninum* (IgM and IgG), and *Toxoplasma gondii* (IgM and IgG) and a SNAP 4DX (IDEXX) test for *Dirofilaria immitis*,* Ehrlichia canis* and *E*. *ewingii*,* Anaplasma phagocytophilum* and *A*. *platys,* and *Borrelia burgdorferi* were negative.

**Figure 1:**
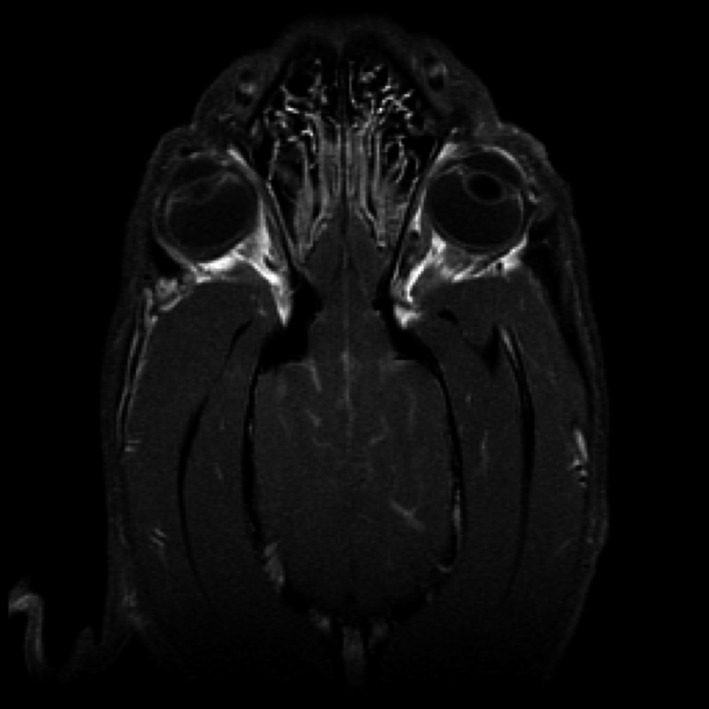
Dorsal plane contrast T1‐weighted MR image of the patient at initial presentation showing meningeal enhancement in the sulci of the cerebral hemispheres.

The patient was anesthetized, and magnetic resonance imaging (MRI; 1.5 T GE) of his brain was performed. Sagittal (T1Wi, T1Wi + gadolinium), transverse (T2Wi, FLAIR, T1Wi, T1Wi + gadolinium, DWi, and ADC map), and dorsal (T2Wi, T1Wi, T1Wi + gadolinium) plane images were obtained. The MRI of the brain showed diffuse contrast enhancement (Magnevist, Bayer) of the leptomeninges (Fig. 1). The differential diagnoses for this severe leptomeningitis were fungal meningitis, round cell neoplasia, and immune‐mediated or other infectious diseases. A CSF sample was collected at the cerebellomedullary cistern. CSF cytology showed numerous round to oval, basophilic, 2–3 *μ*m fungal organisms with an eosinophilic halo and rare narrow‐based budding, most consistent with *Cryptococcus spp*. Complete results of the analysis are available in Table 1. Oral therapy with fluconazole (5 mg/kg BID), prednisone (starting at 0.3 mg/kg BID), and famotidine (0.3 mg/kg BID) was initiated. Prednisone therapy was tapered after 4 days. Two weeks after diagnosis, the physical and neurologic examinations were normal with the exception of granulomatous retinitis of the right eye. One month after diagnosis, the patient's examination was normal. Serial CSF analysis and CSF and serum cryptococcal latex agglutination titers were performed to evaluate treatment over the next year (Table 1). Because of concerns for fluconazole causing rare hepatotoxicity, S‐adenosylmethionine 425 mg was prophylactically administered PO BID.

**Table 1:** Cerebrospinal fluid analysis and cryptococcal agglutination testing results

Time after initial diagnosis	Nucleated Cell Count (cells/*μ*L)	Protein (mg/dL)	Cytology	CSF Cryptococcal agglutination titer	Serum Cryptococcal agglutination titer
0	324	379	40% nondegenerate neutrophils	Positive	n/a
			29% small lymphocytes		
			28% monocytoid		
			3% eosinophils		
			Round to oval fungal 2–3 *μ*m organisms with eosinophilic halo		
2 month	28	111	51% small lymphocytes	1:32	n/a
			31% large monocytoid cells		
			18% eosinophils		
			Macrophages contain degenerating *Cryptococc*us sp. organisms		
5.5 month	17	77	21% eosinophils	1:64	1:128
			35% mononuclear cells		
			44% lymphocytes		
			No microorganisms found		
9 month	43	75	1% nondegenerate neutrophils	Positive	1:16
			57% small lymphocytes		
			33% large monocytoid cells		
			9% eosinophils		
			Rare degenerating organisms contained in monocytoid cells and in background		
11 month	10	51	24% macrophages	n/a	1:32
			31% lymphocytes		
			45% eosinophils		
			Several round to oval fungal organisms with clear capsule found		
13 month	9	35	75% lymphocytes	n/a	1:32
			11% monocytoid cells		
			14% eosinophils		
			No microorganisms are found		
16 month Collected at surgery	0	13	Total of 10 mononuclear cells. A single pink‐staining oval yeast is noted	n/a	Negative
6 year 10 month	0	36	Total of four cells, three macrophages, one small lymphocyte, no organisms or atypical cells	Negative	n/a

Eleven months after diagnosis, the patient represented for an acute onset of a vestibular ataxia and vomiting. On physical examination, the patient demonstrated signs of respiratory disease and a ventral strabismus of the left eye in dorsal recumbency, postural reaction deficits in all limbs, and increased pelvic limb segmental reflexes. Clinical signs localized to the caudal fossa with the potential for a multifocal problem including myelopathy. A recrudescence of cryptococcosis was considered as the highest differential diagnosis. Radiographs of the chest showed a dilated esophagus and aspiration pneumonia. The patient was anesthetized for CSF collection and analysis, which identified an active cryptococcal infection (Table 1).

Treatment included an increased dose of fluconazole (8.6 mg/kg IV BID) and dexamethasone (0.1 and 0.2 mg/kg IV in 48 h). Amphotericin B (Abelcet, Enzon; 1 mg/kg IV) was administered every 48 h. Concurrent treatment of aspiration pneumonia was initiated with: clindamycin (10 mg/kg IV TID), cefotaxime (25 mg/kg IV QID), erythromycin (1 mg/kg IV TID), N‐acetylcysteine (70 mg/kg IV QID), aminophylline (8 mg/kg IV TID), ondansetron (0.2 mg/kg IV BID), ranitidine (2 mg/kg IV BID), metoclopramide (1–2 mg/kg/h), oxygen supplementation, and parenteral nutrition. The patient improved over 14 days with the return of a normal neurologic examination, resolved aspiration pneumonia and resolving megaesophagus. He was released with instructions for continued amphotericin B deoxycholate (ABD) for a total of 12 treatments by the following protocol: 25 mg of ABD mixed with 1 L of 0.45% NaCl and 2.5% dextrose and administered subcutaneously 2–3 times per week.

Over the course of treatment, the patient developed presumptive sterile abscesses at his injection sites over the dorsum. Tramadol (1.7 mg/kg PO BID) was added for discomfort. These swellings resolved without further treatment. Following the 11th treatment, the patient developed an elevated rectal temperature (unknown degree) and lethargy. The treatment series was discontinued.

Two weeks after relapse, the patient demonstrated a spastic tetraparesis and general proprioceptive ataxia with decreased postural reactions in all limbs, which localized to the cranial cervical spinal cord (C1–C5 myelopathy). Radiographs and MRI were offered, but conservative treatment with rest and a change in medications was elected. Due to concerns of relapse on fluconazole, his treatment was changed to itraconazole (6.9 mg/kg PO BID).

**Figure 2:**
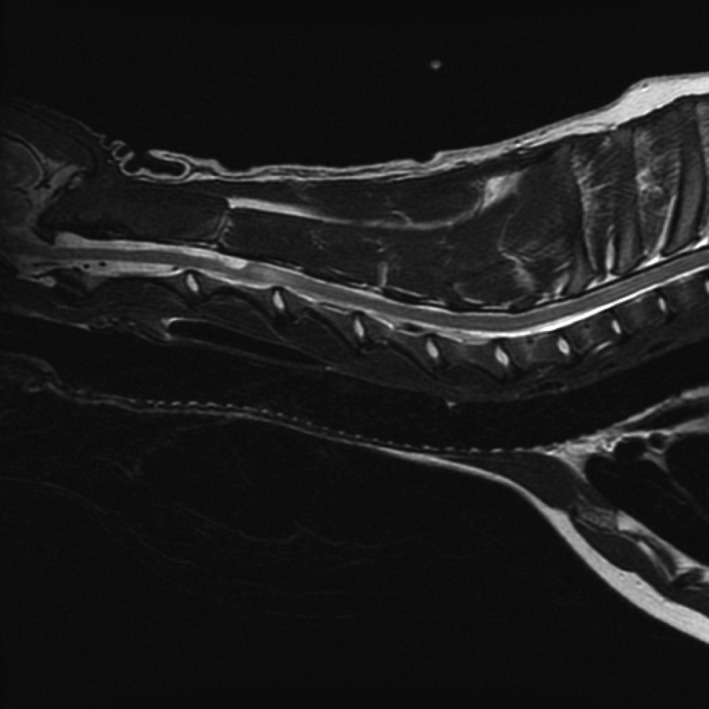
Sagittal plane T2‐weighted MR image of the patient after recovery from relapse showing the widened dorsal subarachnoid space at C2‐C3 with associated intramedullary hyperintensity caudal to the diverticulum.

Three weeks after relapse, the patient had persistent signs of a cervical myelopathy and an MRI was performed. Axial (T2Wi, T1Wi, T1Wi + gadolinium), sagittal (T2Wi) plane images, and a single‐shot fast spin‐echo sagittal sequence were obtained and showed a focal subarachnoid dilation dorsal to C2–C3, compressing the spinal cord. There was an intramedullary T2‐weighted hyperintensity from C3 to C5 (Fig. 2) with no contrast enhancement. The diagnosis was an arachnoid diverticulum with secondary edema or early/developing syringohydromyelia. CSF was collected for analysis, fungal culture, and cryptococcal antigen titer, which were negative. Cytology showed persistent inflammation. Prednisone (0.7 mg/kg PO BID) and omeprazole (0.7 mg/kg PO BID) therapies were initiated. The antifungal medication was changed to fluconazole due to its superior penetration of the blood–brain barrier and lower cost. Prednisone was tapered over 3 weeks to 20 mg (0.7 mg/kg) in the morning and 10 mg (0.3 mg/kg) in the evening.

Three months after relapse, the patient's ataxia continued to wax and wane. The decision was made to address the lesion surgically. The patient underwent a C2–C3 dorsal laminectomy. The meninges were distended at this site with a gray‐blue appearance. A sample of fluid was collected from this distention and submitted for cytology. A 2‐cm incision was made into the dura at C2–C3. The dura was sutured to the aponeurosis of the rhomboideus muscle, and a fat graft followed by a gel foam layer was placed. The incision was closed routinely. One fungal organism was identified via cytological examination of the sample. A second round of 10 treatments of ABD was administered via the subcutaneous protocol outlined above. A urinalysis and serum creatinine level were checked prior to each treatment. At no time were abnormalities noted.

One year postoperatively, the patient showed a mild pelvic limb proprioceptive ataxia and increased patellar reflexes. Hematologic analysis was normal, and an increased ALT (178; normal 5–107 U/L) was evident on a biochemistry panel. At a follow‐up examination 2 years after dorsal laminectomy, the patient's examination was unchanged. Fluconazole and S‐adenosylmethionine were continuously administered with no changes in hematology or biochemistry panels. Due to increasing financial cost, fluconazole was discontinued 4 years after surgery.

At the age of 7 years and 9 months, the patient presented to Penn Vet for a right pelvic lameness. He was stable from previous examinations, but was lame on his right pelvic limb. Pain was localized to the distal femur. Hematology was normal, and a mild elevation in alkaline phosphatase (209, normal 20–155 U/L) was identified on a biochemistry panel. Radiographs of the limb identified a sclerotic area in the right distal femur. A bone biopsy and spinal tap were performed under general anesthesia. The CSF results were normal. The biopsy results identified a neoplasm, most consistent with osteosarcoma. Bone and fungal cultures, and cryptococcal CSF titer were negative. Treatment with a nonsteroidal anti‐inflammatory medication and tramadol was initiated. The patient was euthanized 3 months after the cancer diagnosis after developing changes in the eye consistent with uveitis and significant limb pain. No postmortem was available.

## Background

*Cryptococcus sp*. is a yeast‐like fungus that has been reported to cause disease in dogs, other veterinary species (cats, ferrets, horses, goats, sheep, cattle, dolphins, birds, and koalas) and people. The most common species to cause infection are *Cryptococcus neoformans* and *gattii* (formerly *neoformans* var. *gatii*) 1. Ubiquitous in nature in the filamentous form, upon infection in dogs, this fungus converts to an encapsulated form 2. The polysaccharide capsule provides protection from host immunity and is a key feature for diagnosis 3. The incidence of cryptococcosis in dogs with meningoencephalitis has been reported to be 5.6% 4. Most dogs are young at the time of diagnosis (<4 years of age), large dog breeds are overrepresented, and infection has been associated with outdoor activity 2. In small animals, the route of infection is likely through the nasal passage, gaining access to the CNS through the cribriform plate 5.

Clinical signs are associated with the lesion location in the CNS and can include head tilt, nystagmus, para‐ or tetraparesis/plegia, ataxia, circling, or seizures. Fundic examination may reveal granulomatous chorioretinitis, hemorrhage, or optic neuritis (seen in 40% of infected dogs) 6.

Typical MRI findings in the brain of dogs with CNS cryptococcosis are multifocal parenchymal lesions or are isolated to diffuse meningeal lesions. Parenchymal lesions are hyperintense on T2‐weighted images and iso‐ to hypointense on T1‐weighted images. Meningeal enhancement is common and can be severe 5.

Organisms may be identified with CSF cytologic analysis for a definitive diagnosis. Results of CSF analysis can show various degrees of inflammation and cell types but most show an elevated protein level. In cases where the organism is suspected but not identified, a latex agglutination assay can identify capsular antigen with a sensitivity of 92% to 98% and specificity of 98% 7. This can be performed on CSF or serum, but CSF titer may be higher in dogs with CNS infection. Titer results can be tracked with serial CSF or serum titers; however, testing of CSF is recommended over serology as false negatives can occur in the latter 8.

Treatment options include systemic antifungal therapy with either single‐drug or multidrug protocols including fluconazole, ABD, ABL, flucytosine, or itraconazole with no clear superior protocol in the literature. One study found an improved 10‐day survival in dogs that received glucocorticoids within that period of time 5. Treatment is continued until resolution of clinical signs and is recommended until titers become undetectable 9.

Prognosis is quite variable. Many factors including severity of clinical signs, immune status of the patient, infective species, and treatment options influence the overall prognosis. One study of 21 dogs with cryptococcal infection of the CNS found the median survival time from diagnosis was 7 days (0–3680 days). For those treated with antifungal medication, the MST increased to 12 days. Dogs surviving 4 days had an improved prognosis and MST of 190 days. Seven of 11 dogs were treated with a single azole drug, and in four of the 11 dogs, a combination of ABL/ABD and either azole or flucytosine was used. There was no difference in survival between these dogs 5. Long‐term successful treatment was 55% in another study of 11 dogs with those dogs receiving ABD in combination with either an azole medication or flucytosine 9.

Predictors of a positive outcome in these patients include young age and a short duration of clinical signs. An altered mental status is associated with a poor prognosis 5. Sporadic case reports in a vast array of patients with multiple infectious species have demonstrated a corresponding variety of treatment options and thus prevented conclusions to be drawn regarding specific treatment prognosis in small animals. The literature of the condition in people is similarly distributed with a single recent consensus statement. The recommendation in people is a combination therapy with the fungicidal amphotericin B and flucytosine for 6–10 weeks or amphotericin B and flucytosine for 2 weeks followed by fluconazole for 10 weeks to 12 months 10.

Arachnoid diverticula, previously mistermed arachnoid cysts, are CSF‐filled outpouchings in the subarachnoid space with no epithelial lining and therefore by definition not cysts 11. The exact etiology of arachnoid diverticula is unknown, but in people, they have been shown to be associated with trauma, myelography, arachnoiditis, hemorrhage, or spinal surgery 12. In animals, theories for development include congenital formation, or secondary to adhesive arachnoiditis incited by bacterial or viral infection, hemorrhage, surgery, myelography, intervertebral disk disease, trauma, and degenerative disease. Scar formation results in blockage of fluid flow 13. The expanded diverticulum can cause spinal cord compression and destruction of local parenchyma or cause further disruption of CSF flow and the formation of syringomyelia 12. Surgical decompression and marsupialization have been shown to have good long‐term results for diverticula although this does not always prevent reformation 14. A retrospective study of 122 cases of arachnoid diverticulum identified concurrent CNS disease in 26 dogs, one of which was diagnosed with a myelitis of unknown origin. Two dogs with a previous history of steroid responsive meningitis‐arteritis developed cervical arachnoid diverticula 15. Other concurrent CNS diseases included the following: vertebral malformations, intervertebral disk extrusion or protrusion, atlantoaxial instability, vertebral canal stenosis attributable to articular process hypertrophy, and fibrocartilaginous embolism. Diverticulum formation has also been reported secondary to a porcupine quill foreign body 16.

## Discussion

This is a report of successful treatment of cryptococcal meningitis followed by a diagnosis of an arachnoid diverticulum. The patient was a typical age and size for fungal infection, with MRI findings consistent with cryptococcosis. The meningeal enhancement pattern followed by identification of cryptococcal organisms in the CSF provided a definitive diagnosis. The species was not determined in this case, but determination of species as well as molecular genotype should be considered to aid in the understanding of pathogenesis and for epidemiologic study.

Treatment with a single agent, fluconazole, eliminated clinical signs for 1 year before recrudescence. The decision to use a single‐agent therapy followed the case report of successful management in a similar case and was elected to avoid the risk of renal toxicity with other therapeutic options 8. Although the drug is mainly fungistatic, it has been shown to have fungicidal properties against *Cryptococcus* species and good penetration of the blood–brain barrier.

At the time of the recrudescence, a second agent, liposomal amphotericin B, was successful in controlling signs with minimal side effects. There were no signs of renal toxicity during treatment, but the treatment was discontinued before reaching the goal of a total cumulative dose of 10 mg/kg. The patient developed an elevated rectal temperature after the eleventh treatment. Fever and uncontrolled tremor are also noted to occur in people during treatment and are colloquially termed the “shake and bake.” Fever, chills, nausea, and vomiting are expected side effects of the medication 17. The second round of ABD likely ensured the control of the infection, and again no sign of renal toxicity was recorded. Treatment with fluconazole continued for 5 years during which no liver dysfunction nor signs of recurrence were reported and titers remained negative. Treatment was discontinued due to increasing cost of prescription; however, given the lack of adverse effects could have been continued.

The authors’ current recommendation for small animal patients diagnosed with CNS cryptococcosis is treatment with either the subcutaneous ABD protocol 2–3 times per week or the intravenous ABL protocol to reach a total dose of 10–20 mg/kg in combination with oral fluconazole for at least 6 months. Serial serum titers are followed on a monthly basis. Once the titer reaches an undetectable level for 2 consecutive months, discontinuation of fluconazole is considered with follow‐up serum titers 1–2 months post‐treatment.

One of the important questions raised by this case is that of the pathogenesis of the arachnoid diverticulum. The patient's initial examination findings did not localize to his cervical spinal cord, and thus, only a brain MRI was performed. Imaging findings were consistent with the lesion localization. Therefore, further studies of the CNS were not performed at that time. The possibilities however are few: A congenital diverticulum existed that was exacerbated by the process of diagnosis and infection or the diverticulum formed as a result of either the infection/inflammation or trauma of the repeated CSF collection. Other potential causes of arachnoid diverticula formation in this case included a traumatic cause (although no trauma was reported) or an underlying vertebral instability unrelated to the infection.

A recent case series describes six previously healthy people of a cohort of 26 diagnosed with cryptococcal meningoencephalitis that developed spinal arachnoiditis 18. Patients in this study similarly underwent 3–5 lumbar punctures, and it was theorized that inflammation, both during infection and postinfection, created the environment to allow adhesions to form in the subarachnoid space. Other reviews of human literature point to trauma or surgery (as well as inflammation) as the inciting cause for development 19, 20, 21. However, there is little to no evidence and only vague references to anecdotes regarding lumbar or cisternal taps causing arachnoid diverticula. In fact, in one study conducted to create a model of human spinal arachnoiditis, four control dogs underwent three cisternal taps over 1 year and did not develop subarachnoid changes. Four dogs that received a blood‐contrast medium mixture injected into the subarachnoid space, developed arachnoiditis, fibrosis, and adhesions, highlighting that the inflammation created by more noxious substances in CSF may play a key role in development 22.

For this case, and for others in the future, it is important to note that progression or formation of arachnoid diverticula is a possible cause of developing spinal cord signs.

## Concluding Remarks

This case represents the first report of an arachnoid diverticulum diagnosis following cryptococcal meningitis in a dog. Interesting questions remain the following: Was there a congenital malformation and progression of disease postinfection or did the infection set up the environment for adhesive arachnoiditis? Is the trauma of repeated cerebrospinal fluid collection alone a root cause of arachnoid diverticula formation? If signs localizing to the cervical spinal cord develop after diagnosis and treatment, one must consider an arachnoid diverticulum formation in addition to fungal granuloma and other more routine causes. Cryptococcal meningitis is a rare disease, but future work should be aimed at determining an optimal treatment protocol based on a larger study population and determining treatment endpoints using serum antigen titers. Future studies can also aim to answer whether cisternal or lumbar puncture can incite the progression or formation of arachnoid diverticula.

## Authorship

EG: analyzed and interpreted the patient data, designed and wrote the manuscript. JP: contributed to patient care and wrote the manuscript. All authors: read and approved the final manuscript.

## Conflict of Interest

None declared.
